# *In vitro* interleukin 4 and interferon-gamma production by mononuclear cells from atopic dermatitis patients

**DOI:** 10.1155/S0962935193000584

**Published:** 1993

**Authors:** Serge Well, Friedhelm Diel

**Affiliations:** Department of Nutrition Fachhochschule Fulda FB HE Marquardstr. 35 Fulda 36039 Germany

## Abstract

In order to elucidate further the possible role of specific cytokines in the pathogenesis of atopic dermatitis (AD) the *in vitro* production of interleukin 4 (IL-4) and interferon-gamma (IFN-γ) in patients with severe atopic dermatitis (n = 4) was compared with that in a group of non-atopic healthy controls. Overall IL-4 production by PHA- and PWM-driven PBMNCs was increased in controls during the first 48 h in culture. Addition of interleukin 2 (IL-2) into parallel cultures generated an insignificant (p > 0.05) increase in IL-4 production in AD patients compared with that from controls. IFN-γ production by PWM-stimulated PBMNCs was markedly decreased in AD patients compared with controls (p < 0.01). Addition of IL-2 (250 U/ml) to parallel cultures failed to restore IFN-γ production in AD patients. Finally, no IL-4 or IFN-γ activity could be detected in any of the sera. In conclusion, the data suggest a possible dysregulation of cytokine production in at least a subgroup of AD patients, with an impaired capacity to secrete IFN-γ, but a partially intact IL-4 generating capacity.


Mediators of Inflammation 2, 411–415 (1993)

### *In vitro* interleukin 4 and interferon-gamma production by mononuclear cells from atopic dermatitis patients

**Serge Well and Friedhelm Diel**[CA]

Department of Nutrition, Fachhochschule Fulda, FB HE, Marquardstr. 35, 36039 Fulda, Germany

[CA] Corresponding Author

**IN order to elucidate further the possible role of specific cytokines in the pathogenesis of atopic dermatitis (AD) the *in vitro* production of interleukin 4 (IL-4) and interferon-gamma (IFN-γ) in patients with severe atopic dermatitis ($n = 4$) was compared with that in a group of non-atopic healthy controls. Overall IL-4 production by PHA- and PWM-driven PBMNCs was increased in controls during the first 48 h in culture. Addition of interleukin 2 (IL-2) into parallel cultures generated an insignificant ($p > 0.05$) increase in IL-4 production in AD patients compared with that from controls. IFN-γ production by PWM-stimulated PBMNCs was markedly decreased in AD patients compared with controls ($p < 0.01$). Addition of IL-2 (250 U/ml) to parallel cultures failed to restore IFN-γ production in AD patients. Finally, no IL-4 or IFN-γ activity could be detected in any of the sera. In conclusion, the data suggest a possible dysregulation of cytokine production in at least a subgroup of AD patients, with an impaired capacity to secrete IFN-γ, but a partially intact IL-4 generating capacity.**

**Key words:** Atopic dermatitis, Children, *In vitro* interleukin 4 and interferon-γ production

### Introduction

Although atopic dermatitis (AD) is a chronic, pruritic, inflammatory skin disorder first described as a disease entity in the early decades of this century, its etiopathogenesis has not yet been fully elucidated. In addition to genetic disposition, AD is influenced by many environmental factors. Numerous humoral and cellular immunological abnormalities have been reported in AD,[1] including high IgE levels, increased numbers of IgE-Fc receptor (FcεR) bearing lymphocytes, and cellular dysfunctions in T-cells. In the murine system[2,3] as well as in humans[4] T-helper cells can be divided into two subsets ($TH_1$ and $TH_2$) on the basis of the cytokines they produce: $TH_1$ cells secrete interleukin 2 (IL-2) and interferon-gamma (IFN-γ) and $TH_2$ cells secrete interleukin 4 (IL-4) and interleukin 5 (IL-5). Evidence has accumulated that the human IgE response is mainly regulated by the relative production of the reciprocally active cytokines IL-4 ($TH_2$ system) and IFN-γ ($TH_1$ system).[5–8] Both recombinant and natural IL-4 induce IgE synthesis by peripheral blood mononuclear cells (PBMNCs) from atopic and healthy donors.[9–11] IFN-γ down-regulates this IL-4 induction of IgE synthesis *in vitro*.[5,12–14] It has been suggested that in atopic individuals allergens may 'select' T-helper cells with peculiar profiles of cytokine production (high IL-4 and low IFN-γ), thus contributing to the pathogenesis of IgE-mediated disorders.[6] Only a few studies have investigated the simultaneous production of IL-4 and IFN-γ *in vitro* using activated PBMNCs cultures from atopic donors.[15–17] The results have been in part controversial with respect to IL-4[15–17,18] and IFN-γ[16,17,19,20] production. These data prompted the authors to examine whether IL-4 and IFN-γ activity could be detected in the supernatants (SN) of plant lectin-stimulated (PHA and PWM) PBMNC cultures from AD patients with moderate to severe disease activity using commercially available ELISAs.[21] In order to investigate the *in vitro* effect of IL-2, a T-cell growth factor described as being required for IL-4 production[22,23] as well as capable of mediating IFN-γ generation,[24] exogeneous rhIL-2 was added to parallel PBMNC cultures. We finally investigated whether PBMNCs from atopic patients with severe disease scores produced altered levels of IL-4 and IFN-γ compared with those from controls.

### Materials and Methods

*Subjects:* Four atopic patients (all male; mean age 12 years, range 9–14 years) were selected on the basis of a positive history for allergies. They all suffered from atopic dermatitis as defined by the criteria of Hanifin and Rajka,[25] allergic rhinitis and mild or severe allergic asthma, and had elevated serum total IgE levels (mean 4714 IU/ml, range 222–10920 IU/ml) using the Phadezym

© 1993 Rapid Communications of Oxford Ltd

IgE Prist test (Pharmacia Biosystems, Freiburg, Germany). Three of four atopic patients had acute exacerbation of AD, with severe episodes of itching, paralleled by nocturnal asthmatic attacks, requiring various therapeutic regimens including topical emollients, ambulant photo-balneotherapy and/or elimination diets. The control group consisted of four healthy individuals (all male; mean age 20 years, range 8–29 years) with a negative history of atopy; their total serum IgE levels were <50 IU/ml; no elevated specific IgE levels to the most common allergens were detected in the sera using the Phadezym RAST test (Pharmacia).

*Reagents:* RPMI 1640 medium, supplemented with 300 mg/ml L-glutamine, 100 U/ml penicillin, 100 mg/ml streptomycin, 10% heat-inactivated (30 min, 56°C) foetal bovine serum, and 25 mM HEPES buffer, phytohaemagglutinin (PHA), pokeweed mitogen (PWM), and recombinant human interleukin 2 (rhIL-2) were all obtained from Sigma Chemie, Deisenhofen, Germany. Ficoll–Hypaque® was obtained from Pharmacia, Freiburg, Germany.

*Cells and cultures:* PBMNCs were isolated from heparinized peripheral blood by centrifugation on a density gradient of Ficoll–Hypaque,[26] extensively washed and resuspended at $10^6$ cells/ml of complete medium. 200 μl of cell suspension/well were cultured with or without PHA or PWM (at final concentrations of 5.0 μg/ml), either in the presence or absence of IL-2 (250 U/ml) in 96-well, flat-bottomed, polystyrene microtitre plates (Corning®, Sigma Chemie) at 37°C, 95% humidity, and 10% $CO_2$ in air for 48 h. At 24 h and 48 h after initiation the culture supernatants (SN) were harvested by centrifuging the samples at 350 × *g* for 10 min at room temperature. The cell pellets were resuspended in fresh complete RPMI 1640 medium. The SNs were stored in aliquots at −70°C until tested.

*Cytokine assays:* IL-4 was determined in the SNs of the PBMNC cultures using a commercially available ELISA (Quantikine®, Biermann Diagnostics, Bad Nauheim, Germany). The same technique was used for measuring IL-4 in the donors' sera, which were frozen at −20°C 1 h after collection and centrifugation. The calculated lower limit of detection of this ELISA is 2–3 pg/ml with a confidence of 96%. The SNs and sera were assayed for IFN-γ with a commercially available ELISA for human IFN-γ (HBT, Biermann Diagnostics). According to the manufacturer 1 unit of natural human IFN-γ corresponds to between 50 and 100 pg, depending on the batch. Each sample was measured in duplicate unless stated otherwise.

*[$^3$H]-thymidine incorporation assay:* Proliferation of the PBMNCs in culture was assessed by a standard [$^3$H]-thymidine incorporation assay.

*Statistical evaluations:* Differences in levels of IL-4 and IFN-γ between AD patients and controls were analysed by Student's *t*-test. Correlations between cytokine production and IgE serum concentration were calculated by simple linear regression analysis and tested for statistical significance. Owing to the small samples sizes, descriptive rather than confirmatory statistics were intended.

### Results

*IL-4 and IFN-γ in human serum:* No IL-4 or IFN-γ activity could be detected in the non-concentrated sera from AD patients or healthy controls with the above described ELISA techniques (IL-4 < 3 pg/ml resp. IFN-γ < 1 pg/ml).

*Time course of cytokine production in PBMNC cultures:* IL-4 and IFN-γ were not spontaneously secreted by PBMNCs from either group. The time courses (over 48 h) of cytokine production by PBMNCs from AD patients after addition of PHA (5.0 μg/ml) or PWM (5.0 μg/ml) are shown in Fig. 1.

*IL-4 and IFN-γ production in PBMNC cultures (at 24 h):*

*Effects of PWM stimulation:* PWM (5.0 μg/ml)-induced IL-4 production (at 24 h) by PBMNCs from AD patients was not increased significantly compared with controls. PWM-induced IFN-γ generation (at 24 h) in AD patients (mean 5.5 ± 5.4 U/ml) was decreased significantly ($p$ < 0.01) compared with controls (mean 42.5 ± 12.3 U/ml) (Fig. 2).

*Effects of PHA stimulation.* PHA (5.0 μg/ml)-induced IL-4 production as well as IFN-γ generation (at 24 h) by PBMNCs from AD patients

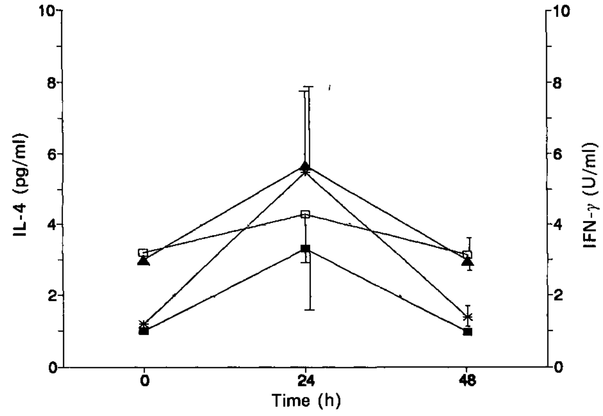

FIG. 1. Kinetics of *in vitro* IL-4 and IFN-γ production by PBMNCs from AD patients ($n$ = 4) with increased serum IgE levels; $10^6$ PBMNCs/ml of complete RPMI 1640 medium were stimulated with 5 μg/ml of PHA or 5 μg/ml of PWM. After 24 and 48 h, SNs were harvested and assayed for IL-4 (PHA = plain triangles; PWM = open squares) and IFN-γ (PHA = plain squares; PWM = stars) activity. Each sample was measured in duplicate. Values are expressed as means ± S.E.M.

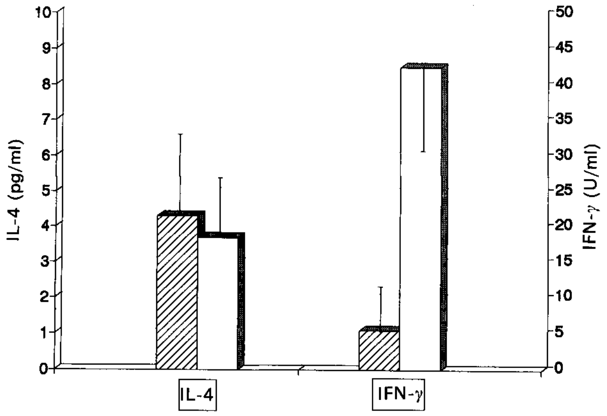

FIG. 2. Cytokine production *in vitro* by PWM-stimulated PBMNCs. PWM-induced IL-4 generation by PBMNCs from AD patients (hatched bars) (at 24 h) was not significantly increased ($p \leq 0.05$) compared with that from controls (open bars). PWM-induced IFN-γ generation (at 24 h) in AD patients (mean 5.5 ± 5.4 U/ml) was significantly decreased ($p < 0.01$) compared with that in controls (mean 42.5 ± 12.3 U/ml). Values are expressed as means ± S.E.M.

did not differ significantly from controls (data not shown).

*Effects of IL-2 supplementation.* In order to investigate the IL-2 requirement for IL-4 generation, rhIL-2 (250 U/ml) was added with PWM or PHA. IL-2 supplementation did not increase IL-4 production significantly in AD patients and controls. We further investigated whether IL-2 can restore the impaired IFN-γ production in AD patients. No significant difference was observed between IFN-γ secretion (at 24 h) upon PWM plus IL-2 stimulation compared with PWM stimulation alone (Fig. 3) or PHA plus IL-2 stimulation compared with PHA stimulation alone.

*Correlation between cytokine production and IgE serum concentration:* A significant positive correlation existed between *in vitro* PHA- ($r = 0.85$, $p < 0.05$) and PWM- ($r = 0.98$, $p < 0.00005$) induced IFN-γ secretion and total IgE serum concentration in AD patients; no significant correlation was observed between PHA or PWM-induced IL-4 secretion and total IgE serum concentration (data not shown).

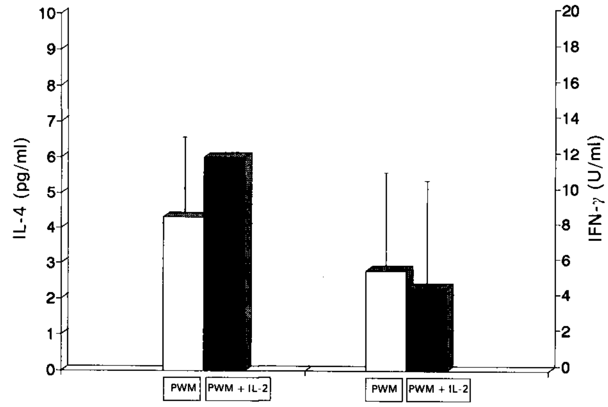

FIG. 3. Effects of IL-2 addition on PWM-induced cytokine production. Addition of IL-2 (250 U/ml) to PWM-stimulated PBMNCs (black bars) of AD patients did not significantly increase IL-4 production (at 24 h) compared with that of cultures grown with PWM alone (open bars). No significant difference was observed between IFN-γ secretion (at 24 h) upon PWM plus IL-2 stimulation compared with PWM stimulation alone. Values are expressed as means ± S.E.M.

### Discussion

In this study the *in vitro* production of IL-4 and IFN-γ by PBMNCs from AD patients was compared with that of healthy controls. In agreement with other published data, neither IL-4[16] nor IFN-γ[19] could be detected in any of the sera. This is in contrast to data published by Matsumoto *et al.*,[27] where serum concentrations of IL-4 could be measured in children with allergic diseases as well as in non-allergic controls. Differences in the IL-4 assays used might partly account for these discrepancies.

More importantly, *in vitro* PWM-induced IFN-γ production by PBMNCs from AD patients was significantly lower compared with controls (Fig. 2). An accepted view is that the elevated serum IgE levels in atopic individuals are a consequence of imbalances between IL-4-producing and IFN-γ-producing helper T-cells.[28] Parronchi *et al.*[29] suggested that the high amounts of IL-4 without simultaneous IFN-γ production by allergen-specific TH cells induces the formation of IgE antibodies. The defective IFN-γ production in AD patients observed here might not be able to successfully down-regulate IL-4-induced IgE synthesis *in vivo* in these individuals, whereas high IFN-γ levels in healthy controls might successfully antagonize the IL-4-induced IgE synthesis. However, as unseparated PBMNCs were used as the cell source in this study the potential contribution of monocytes to the measured IFN-γ production is difficult to assess. Del Prete *et al.*[30] reported defective IFN-γ production in the hyper IgE syndrome. A severe deficiency in IFN-γ production by PBMNCs from AD patients was observed after PHA stimulation[31,32] as well as concanavalin stimulation.[33] In this study defective IFN-γ production in AD patients has been shown by using PWM, another lectin that does not bypass the transmembrane signalling when stimulating PBMNCs.

As IL-2 can mediate mitogen-induced IFN-γ production,[24] the authors investigated whether stimulation with mitogen plus IL-2 could restore decreased IFN-γ production by PBMNCs *in vitro*. Supplementing PBMNCs from AD patients with up to 250 U/ml of IL-2 failed to restore PWM- or PHA-induced IFN-γ production. At this final concentration even IL-2 had a depressive effect on IFN-γ production. Taken together, these data suggest that the reported defective IL-2 production[34] in AD patients is possibly not responsible for the decreased IFN-γ production by PBMNCs.

The observed positive correlation in AD patients between *in vitro* IFN-$\gamma$ production and total serum IgE is in apparent conflict with the view that IFN-$\gamma$ down-regulates IgE production and in contrast with another report.[31]

The data presented here do not demonstrate a significant difference between PHA/PWM-induced IL-4 production in AD patients and controls (Fig. 2). Supplementation with IL-2 (250 U/ml) failed to induce a significant increase in IL-4 generation from PHA/PWM-stimulated PBMNCs from AD patients. Seder *et al.*[35] have shown that the frequency of IL-4 producing cells is very low among circulating antigen-primed T-cells; furthermore several groups recently reported evidence for the accumulation and expansion of T-lymphocytes producing high amounts of IL-4 in lesional skin, whereas T-lymphocytes from peripheral blood were poor IL-4 producers.[36,37] Further investigations should compare the cytokine profiles from lesional skin cells and the data obtained here from peripheral blood cells in the same AD individuals. However it is remarkable that, despite the now accepted view that IFN-$\gamma$ and IL-4 predominantly act at a local level, a highly significant decreased *in vitro* IFN-$\gamma$ production can be observed among peripheral blood MNCs.

In contrast to IL-4, no correlation between IFN-$\gamma$ production and cell proliferation was observed. However, Patarca *et al.*[38] demonstrated that activation of $TH_1$ cells does not necessarily result in IFN-$\gamma$ production.

In conclusion, although the number of investigated individuals is small, we could demonstrate a defective *in vitro* IFN-$\gamma$ production in PBMNCs from patients with mild to severe forms of AD and moderate to high total serum IgE concentrations, whereas no significant difference in IL-4 secretion was observed in these patients when compared with controls.

### References

1. Leung DYM, Geha RS. Immunoregulatory abnormalities in atopic dermatitis. *Clin Rev Allergy* 1986; **4**: 67–86.
2. Mosmann TR, Coffman RL. Two types of mouse helper T cell clones. *Immunol Today* 1987; **8**: 223–37.
3. Coffmann RL, Seymour BWP, Lebman DA *et al.* The role of helper T cell products in mouse B cell differentiation and isotype regulation. *Immunol Rev* 1988; **102**: 5-28.
4. Wierenga EA, Snoek M, De Groot C *et al.* Evidence for compartmentalization of functional subsets of CD4$^+$ T lymphocytes in atopic patients. *J Immunol* 1990; **144**: 4651–4656.
5. Pène J, Rousset F, Brière F *et al.* IgE production by normal human lymphocytes is induced by interleukin 4 and suppressed by interferons $\gamma$ and $\alpha$ and prostaglandin $E_2$. *Proc Natl Acad Sci USA* 1988; **85**: 6880–6884.
6. Romagnani S. Regulation and deregulation of human IgE synthesis. *Immunol Today* 1990; **11**: 316–321.
7. de Vries JE, Gauchat J-F, Aversa GG, Punnonen J, Gascan H, Yssel H. Regulation of IgE synthesis by cytokines. *Curr Opin Immunol* 1991; **3**: 851–858.
8. Chrétien I, Pène J, Brière F, de Waal Malefijt R, Rousset F, de Vries JE. Regulation of human IgE synthesis. I. Human IgE synthesis *in vitro* is determined by the reciprocal antagonistic effects of interleukin 4 and interferon-$\gamma$. *Eur J Immunol* 1990; **20**: 243–251.
9. Pène J, Rousset F, Brière F *et al.* IgE production by normal B cells induced by alloreactive T cell clones is mediated by IL-4 and suppressed by IFN-$\gamma$. *J Immunol* 1988; **141**: 1218–1224.
10. Del Prete G, Maggi E, Parronchi P *et al.* IL-4 is an essential factor for the IgE synthesis induced *in vitro* by human T cell clones and their supernatants. *J Immunol* 1988; **140**: 4193–4198.
11. Claasen JL, Levine AD, Buckley RH. Recombinant human IL-4 induces IgE and IgG synthesis by normal and atopic donor mononuclear cells. *J Immunol* 1990; **144**: 2123–2130.
12. Pène J, Rousset F, Brière F *et al.* IgE production by normal human B cells induced by alloreactive T cell clones is mediated by IL-4 and suppressed by IFN-$\gamma$. *J Immunol* 1988; **141**: 1218–1224.
13. Romagnani S, Del Prete G, Maggi E *et al.* Role of interleukins in induction and regulation of human IgE synthesis. *Clin Immunol Immunopathol* 1989; **50**: S13–S23.
14. Vercelli D, Jabara HH, Lauener RP, Geha RS. IL-4 inhibits the synthesis of IFN-$\gamma$ and induces the synthesis of IgE in human mixed lymphocyte cultures. *J Immunol* 1990; **144**: 570–573.
15. Paliard X, de Waal Malefijt R, Yssel H *et al.* Simultaneous production of IL-2, IL-4, and IFN-$\gamma$ by activated human CD4$^+$ and CD8$^+$ T cell clones. *J Immunol* 1988; **141**: 849–855.
16. Rousset F, Robert J, Andary M *et al.* Shifts in interleukin-4 and interferon-$\gamma$ production by T cells of patients with elevated serum IgE levels and the modulatory effects of these lymphokines on spontaneous IgE synthesis. *J Allergy Clin Immunol* 1991; **87**: 58–69.
17. Butch AW, Pesando J, Levine AD, McKearn JP, Nahm MH. Cytokine production by T helper cell subpopulations during prolonged *in vitro* stimulation. *Immunol Lett* 1991; **27**: 85–94.
18. Scott MG, Levine AD, Butch AW, Bowen MB, Macke KA, Nahm MH. Production, characterization and use of five monoclonal antibodies to human IL-4. *Lymphokine Res* 1990; **9**: 81–93.
19. Kapp A, Gillitzer R, Kirchner H, Schöpf E. Production of interferon and lymphoproliferative responses in whole blood cultures derived from patients with atopic dermatitis. *Arch Derm Res* 1987; **279**: S55–S58.
20. Wehrmann W, Reinhold U, Pawalec G, Wernet P, Kreysel H-W. *In vitro* generation of IFN-gamma in relationship to *in vivo* concentrations of IgE and IgG subclasses and $Fc_{\epsilon}R_{I}$/CD23-positive circulating lymphocytes in patients with severe atopic dermatitis. *Acta Derm Venereol (Stockh)* 1989; **Suppl 144**: 127–130.
21. Well S, Paetzold M, Choné K, Diel F. Interleukin-4 in mononuclear cells and histamine release in peripheral blood from an atopic patient *in vitro*. *Agents Actions* 1992; **Special Issue C**: C298–C301.
22. Le Gros G, Ben-Sasson SZ, Seder R, Finkelman FD, Paul WE. Generation of interleukin 4 (IL-4)-producing cells *in vivo* and *in vitro*: IL-2 and IL-4 are required for *in vitro* generation of IL-4-producing cells. *J Exp Med* 1990; **172**: 921–929.
23. Ben-Sasson SZ, Le Gros G, Conrad DH, Finkelman FD, Paul WE. IL-4 production by T cells from naive donors. IL-2 is required for IL-4 production. *J Immunol* 1990; **145**: 1127–1136.
24. Vilcek J, Henriksen-Destefano D, Siegel D, Klion A, Robb RJ, Le J. Reguation of IFN-gamma, induction in human peripheral blood cells by exogeneous and endogeneously produced interleukin 2. *J Immunol* 1985; **135**: 1851–1855.
25. Hanifin JM, Rajka G. Diagnostic features of atopic dermatitis. *Acta Derm Venereol (Stockh.)* 1980; **Suppl 92**: 44–47.
26. Bøyum A. Isolation of mononuclear cells and granuloctes from human blood. *Scand J Clin Lab Invest* 1968; **21**: 77–89.
27. Matsumoto T, Miike T, Yamaguchi K, Murakami M, Kawabe T, Yodoi J. Serum levels of soluble IL-2 receptor, IL-4 and IgE-binding factors in childhood allergic diseases. *Clin Exp Immunol* 1991; **85**: 288–292.
28. Romagnani S, Del Prete GF, Maggi E *et al.* Role of T cells and T-cell-derived cytokines in the regulation and deregulation of human immunoglobulin E synthesis. *Advances in Allergology and Clinical Immunology*, Proceedings of the XVth Congress of the EAACI, Paris, France, 1992; 9–14.
29. Parronchi P, Macchia D, Piccinni MP *et al.* Allergen and bacterial antigen-specific T-cell clones established from atopic donors show a different profile of cytokine production. *Proc Natl Acad Sci USA* 1991; **88**: 4538–4542.
30. Del Prete G, Tiri A, Maggi E *et al.* Defective *in vitro* production of $\gamma$-interferon and tumor necrosis factor-$\alpha$ by circulating T cells from patients with the hyperimmunoglobulin E syndrome. *J Clin Invest* 1989; **84**: 1830–1835.
31. Reinhold U, Wehrmann W, Kukel S, Kreysel HW. Evidence that defective interferon-gamma production in atopic dermatitis patients is due to intrinsic abnormalities. *Clin Exp Immunol* 1990; **79**: 374–380.
32. Reinhold U, Pawalec G, Wehrmann W, Herold M, Wernet P, Kreysel HW. Immunoglobulin E and immunoglobulin G subclass distribution *in vivo* and relationship to *in vitro* generation of interferon-gamma in patients with severe atopic dermatitis. *Int Arch Allergy Appl Immunol* 1988; **87**: 120–126.
33. Boguniewicz M, Jaffe HS, Izu A *et al.* Recombinant gamma interferon in the treatment of patients with atopic dermatitis and elevated IgE levels. *Am J Med* 1990; **88**: 365–370.
34. Kapp A, Kirnbauer R, Luger TA, Schöpf E. Altered production of immuno-modulating cytokines in patients with atopic dermatitis. *Acta Derm Venereol (Stockh)* 1989; **Suppl 144**: 97–99.
35. Seder RA, Le Gros G, Ben-Sasson SZ, Urban J Jr, Finkelman FD, Paul WE. Increased frequency of interleukin 4-producing T cells as a result of polyclonal priming. Use of a single-cell assay to detect interleukin 4-producing cells. *Eur J Immunol* 1991; **21**: 1241–1247.
36. van der Heijden FL, Wierenga EA, Bos JD, Kapsenberg ML. High frequency of IL-4-producing $CD4^+$ allergen-specific T lymphocytes in atopic dermatitis lesional skin. *J Invest Dermatol* 1991; **97**: 389–394.
37. Sager N, Feldmann A, Schilling G, Kreitsch P, Neumann C. House dust mite-specific T cells in the skin of subjects with atopic dermatitis: frequency and lymphokine profile in the allergen patch test. *J Allergy Clin Immunol* 1992; **89**: 801–810.
38. Patarca R, Wei FY, Iregui MV, Cantor H. Differential induction of IFN-γ gene expression after activation of $CD4^+$ T cells by conventional antigen and Mls superantigen. *Proc Natl Acad Sci USA* 1991; **88**: 2736–2739.

ACKNOWLEDGMENTS. This work was supported in part by a grant from Hessisches Ministerium für Wissenschaft und Kultur. The authors are grateful to Dr Madeleine Ennis, Queen's University of Belfast, for her critical review of the manuscript and to the Allergie Verein in Europa AVE for co-operation in patient management.

**Received 7 July 1993;
accepted in revised form 2 September 1993**
